# Identification and characterization of a 20β-HSDH from the anaerobic gut bacterium *Butyricicoccus desmolans* ATCC 43058[S]

**DOI:** 10.1194/jlr.M074914

**Published:** 2017-04-28

**Authors:** Saravanan Devendran, Celia Méndez-García, Jason M. Ridlon

**Affiliations:** Department of Animal Sciences,* University of Illinois at Urbana-Champaign, Urbana, IL 61801; Carl R. Woese Institute for Genomic Biology,† University of Illinois at Urbana-Champaign, Urbana, IL 61801; Division of Nutritional Sciences,§ University of Illinois at Urbana-Champaign, Urbana, IL 61801

**Keywords:** American Type Culture Collection, 20β-hydroxysteroid dehydrogenase, glucocorticoids, gut microbiota

## Abstract

Members of the gastrointestinal microbiota are known to convert glucocorticoids to androstanes, which are subsequently converted to potent androgens by other members of the gut microbiota or host tissues. *Butyricicoccus desmolans* and *Clostridium cadaveris* have previously been reported for steroid-17,20-desmolase and 20β-hydroxysteroid dehydrogenase (HSDH) activities that are responsible for androstane formation from cortisol; however, the genes encoding these enzymes have yet to be reported. In this work, we identified and located a gene encoding 20β-HSDH in both *B. desmolans* and *C. cadaveris*. The 20β-HSDH of *B. desmolans* was heterologously overexpressed and purified from *Escherichia coli*. The enzyme was determined to be a homotetramer with subunit molecular mass of 33.8 ± 3.7 kDa. The r20β-HSDH displayed pH optimum in the reductive direction at pH 9.0 and in the oxidative direction at pH 7.0–7.5 with (20β-dihydro)cortisol and NAD(H) as substrates. Cortisol is the preferred substrate with *K_m_*, 0.80 ± 0.06 μM; *V_max_*, 30.36 ± 1.97 μmol·min^−1^; *K_cat_*, 607 ± 39 μmol·μM^−1^·min^−1^; *K_cat_*/*K_m_*, 760 ± 7.67. Phylogenetic analysis of the 20β-HSDH from *B. desmolans* suggested that the 20β-HSDH is found in several *Bifidobacterium spp*., one of which was shown to express 20β-HSDH activity. Notably, we also identified a novel steroid-17,20-desmolase-elaborating bacterium, *Propionimicrobium lymphophilum*, a normal inhabitant of the urinary tract.

Studies by Nabarro et al. (1) and Wade et al. (2) reported that rectally infused cortisol in humans resulted in a 100-fold increase in urinary 17-ketosteroids, which was ablated by oral neomycin treatment. Winter and Bokkenheuser (3) and Cerone-McLernon et al. (4) reported that incubation of cortisol with mixed human and rat fecal microbiota resulted in the accumulation of C-19 17β-hydroxy metabolites. These studies first pointed to a bacterial pathway, known as steroid-17,20-desmolase, which converts cortisol into 11β-hydroxyandrostenedione (11β-OHAD). Quantification of steroid-17,20-desmolase activity in decimal dilutions of human and rat fecal samples suggests approximately 10^6^ organisms per gram wet-weight feces (5).

Bokkenheuser et al. (5) isolated the first fecal bacterium in pure culture that expresses steroid-17,20-desmolase and 20α-hydroxysteroid dehydrogenase (HSDH). The gut bacterium was later named *Clostridium scindens* (type strain ATCC 35704) (6). Krafft and colleagues characterized the purified 20α-HSDH and partially-pure steroid-17,20-desmolase from cell extracts of *C. scindens* ATCC 35704 and reported substrate-specificity and cofactor requirements (7, 8). However, the genes encoding these enzymes and the mechanism by which anaerobic bacterial steroid-17,20-desmolase proceeds were unclear. Recent work by Ridlon et al. (9) identified a cortisol-inducible multi-gene operon (*desABCD*) hypothesized to encode bacterial steroid-17,20-desmolase and 20α-HSDH. The *desAB* genes are annotated as “transketolase” N-terminal and C-terminal subunits (9). The two-carbon fragment ketol group transferred via thiamine-pyrophosphate from a sugar phosphate donor to aldose acceptors during transketolation is identical to the side-chain of cortisol, suggesting that steroid-17,20-desmolase perhaps proceeds by a transketolation reaction (9). A cortisol-inducible “Na^+^-dependent melibiose transporter” encoded by the *desD* gene was also identified and hypothesized to encode a cortisol transport protein. Purified recombinant DesC was shown to have substrate-specificity and kinetics consistent with previously purified native 20α-HSDH (7, 8). In these studies, steroids with 20β-hydroxy groups were not substrates for DesC (9).

Steroid-17,20-desmolase activity has also been reported in *Clostridium cadaveris* and *Butyricicoccus desmolans* (formerly *Eubacterium desmolans*) (10, 11). Unlike *C. scindens* ATCC 35704, which elaborates 20α-HSDH, both *C. cadaveris* and *B. desmolans* have been reported to express 20β-HSDH activity (10). Following the report of the *desABCD* operon from *C. scindens* ATCC 35704, genome sequences for *B. desmolans* ATCC 43058 (Bio Project PRJNA223055) and *C. cadaveris* (Bio Projects PRJNA185698, PRJEB17278) became available, prompting us to determine whether these anaerobic bacteria encode the *desAB* genes in addition to an NAD(P)H-dependent dehydrogenase encoding 20β-HSDH.

## MATERIALS AND METHODS

### Bacterial strains and materials

*B. desmolans* ATCC 43058 and *Bifidobacterium scardovii* ATCC BAA-773 were purchased from ATCC. *C. scindens* ATCC 35704 was maintained as −80°C glycerol stocks in our laboratory. *Propionimicrobium lymphophilum* ACS-093-V-SCH5 was obtained from the Culture Collection, University of Götesborg, Sweden. *Escherichia coli* DH5α (turbo) competent cells were from New England Biolabs (Ipswich, MA) and *E. coli* BL21-CodonPlus(DE3) RIPL was purchased from Stratagene (La Jolla, CA). The pET-51b(+) vector was obtained from Novagen (San Diego, CA). Restriction enzymes were purchased from New England Biolabs; QIAprep Spin Miniprep kit was obtained from Qiagen (Valencia, CA). Isopropyl β-D-1-thiogalactopyranoside was purchased from Gold Biotechnology (St. Louis, MO). Strep-Tactin resin was purchased from IBA GmbH (Gottingen, Germany). Steroids were purchased from Steraloids (Newport, RI). Amicon Ultra-15 centrifugal filter units with 30 and 50 kDa molecular-mass cutoffs were obtained from Millipore (Billerica, MA). All other reagents were of the highest possible purity and were purchased from Fisher Scientific (Pittsburgh, PA).

### Steroid conversion experiments

*B. desmolans* ATCC 43058, *B. scardovii* ATCC BAA-773, and *C. scindens* ATCC 35704 were cultivated in supplemented brain heart infusion (BHI) broth, containing 5 g l^−1^ of yeast extract. *P. lymphophilum* ACS-093-V-SCH5 was grown in Gifu anaerobic medium. Steroids were dissolved in methanol and added to the sterilized medium at a concentration of 50 μM, unless otherwise indicated; the concentration of methanol (v/v) per culture was kept at 0.5%. The medium was then seeded with 0.1 ml of a pure bacterial culture and incubated at 37°C for 2 days. After incubation, the products were extracted by vortexing the culture media with 2 vol of ethyl acetate for 1–2 min and then the organic phase was recovered. The organic phase was evaporated under nitrogen gas. The residue was dissolved in 50 μl methanol and analyzed using HPLC (Shimadzu, Japan) equipped with a C-18 analytical column (Capcell Pak c18; Shiseido, Japan). The mobile phase consisted of acetonitrile/water with 0.01% formic acid and the flow rate was maintained at 0.2 ml·min^−1^. A DAD detector was used at a wavelength of 254 nm. Peak retention times and peak areas of samples were compared with standard steroid molecules.

#### RNA isolation and transcriptional analysis.

*B. desmolans* ATCC 43058 was cultivated in BHI broth in the presence or absence of 50 μM cortisol until mid-log phase. Cultures were centrifuged at 16,000 *g* for 5 min and the pellet suspended in RNALater*™* (Ambion) overnight at 4°C followed by centrifugation and storage at −70°C until further processing. Total RNA was isolated using the Ribopure bacteria kit (Ambion) according to the manufacturer’s instructions, including the DNase step. One microgram of total RNA was converted to cDNA using the Advantage RT-for-PCR kit (Clontech) with random hexamer primers. Intergenic PCR was performed using TITANIUM Taq PCR kit (Clontech) with oligonucleotides synthesized by Integrated DNA Technologies, Inc. (Coralville, IA). All primers used in this study are listed in supplemental Table S1. The mRNA start-site was determined by 5′-RACE PCR using the SMARTer RACE PCR kit (Clontech) according to the manufacturer’s instructions with some modifications. RACE PCR products were purified from 1.5% agarose gels by GENECLEAN spin kit according to the manufacturer’s instructions (Bio 101) and cloned into a TA vector. The plasmid DNA was sequenced to identify the mRNA start sequence (W. M. Keck Center for Comparative and Functional Genomics at the University of Illinois at Urbana-Champaign).

#### Gene cloning.

*B. desmolans* ATCC 43058 genomic DNA was extracted using the Fast DNA isolation kit from Mo-Bio (Carlsbad, CA) according to the manufacturer’s protocol. Oligonucleotide primers used for amplifying the 20β-HSDH gene are reported in supplemental Table S1. The coding sequence for 20β-HSDH was amplified using the Phusion high-fidelity polymerase (Stratagene) and cloned into a pET-51b vector using *Bam*HI and *Hin*dIII restriction sites. Recombinant plasmid was transformed into chemically competent *E. coli* DH5α cells via the heat stock method, plated, and grown for 16 h at 37°C on lysogeny broth (LB) agar plates supplemented with ampicillin (100 μg/ml). A single colony from each transformation was inoculated into LB medium (5 ml) containing ampicillin (100 μg/ml) and grown to saturation. The cells were subsequently centrifuged (3,220 *g*, 15 min, 4°C) and plasmids were extracted from the resulting cell pellet using the QIAprep Spin Miniprep kit (Qiagen). The foreign DNA inserts in the recombinant plasmids were sequenced to confirm the correctness of the gene sequence (W. M. Keck Center for Comparative and Functional Genomics at the University of Illinois at Urbana-Champaign).

### Gene expression and protein purification

For protein expression, the correct recombinant plasmids extracted from the *E. coli* DH5α cells were transformed into *E. coli* BL-21 CodonPlus (DE3) RIPL chemically competent cells by the heat shock method and grown overnight at 37°C on LB agar plates supplemented with ampicillin (100 μg/ml) and chloramphenicol (50 μg/ml). After 16 h, five isolated colonies were used to inoculate 10 ml of fresh LB medium supplemented with antibiotics and grown at 37°C for 6 h with vigorous aeration. The precultures were then added to fresh LB medium (1 liter), supplemented with the same antibiotics at the same concentrations, and grown with vigorous aeration at 37°C. At an OD_600_ of 0.3, isopropyl β-D-1-thiogalactopyranoside was added to each culture at a final concentration of 0.1 mM and the temperature was decreased to 16°C. Following 16 h of culturing, cells were pelleted by centrifugation (4,000 *g*, 30 min, 4°C) and resuspended in 30 ml of binding buffer [20 mM Tris-HCl, 150 mM NaCl, 20% glycerol, 10 mM 2-mercaptoethanol (pH 7.9)]. The cell suspension was subjected to four passages through an EmulsiFlex C-3 cell homogenizer (Avestin, Ottawa, Canada) and the cell lysate was clarified by centrifugation at 20,000 *g* for 30 min at 4°C.

The recombinant 20β-HSDH (r20β-HSDH) was then purified using Strep-Tactin® resin (IBA GmbH) as per the manufacturer’s protocol. The recombinant protein was eluted using an elution buffer composed of 20 mM Tris-HCl, 150 mM NaCl, 20% glycerol, 10 mM 2-mercaptoethanol (pH 7.9), and 2.5 mM desthiobiotin. The protein purity was assessed by SDS-PAGE and protein bands were visualized by staining with Coomassie brilliant blue G-250. The protein concentrations were calculated based on the molecular mass (41.3 kDa) and computed extinction coefficient (31.6 × 10^3^ M^−1^·cm^−1^) (12).

### Gel filtration chromatography

Gel filtration chromatography was carried out on Superdex 200 analytical column (GE Healthcare, Piscataway, NJ) attached to Akta FPLC (GE Healthcare) at 4°C. The eluted protein from the Strep-tag column was concentrated and loaded on the column with a buffer composed of 50 mM Tris-Cl, 300 mM NaCl, 10% glycerol, 10 mM 2- mercaptoethanol, and 0.1% Triton X-100 (pH 7.5). The native molecular mass of the protein was calculated by peak retention volume of protein to retention volume of standard proteins. Eluted fractions were visualized by staining with Coomassie brilliant blue G-250. The activity of the enzyme in the eluted fraction was calculated as described below in the standard enzyme assay section.

### The 20β-HSDH enzyme assay

The 20β-HSDH activity was measured aerobically by monitoring the conversion of cortisol into 20β-dihydrocortisol using a continuous spectrophotometric method, monitored by measuring the oxidation of NADH at 340 nM (*ε* = 6,220 M^−1^·cm^−1^). Linearity with respect to time and enzyme concentration was determined. The standard reaction mixture contained 50 mM phosphate buffer (pH 7.5), 50 μM of cortisol, 150 μM NADH, 0.05 μM enzyme, and buffer to a final volume of 0.5 ml. The reaction was started by the addition of the enzyme. The initial velocity of enzyme-catalyzed reactions was plotted against the substrate concentrations, and the kinetic parameters were estimated by fitting the data to the Michaelis-Menten equation by the nonlinear regression method using the enzyme kinetics module in GraphPad Prism (GraphPad Software, La Jolla, CA).

### Determination of optimal pH

The buffers used to study the pH profiling of 20β-HSDH were as follows: 50 mM sodium citrate, 150 mM NaCl, 20% glycerol and 10 mM 2-mercaptoethanol (pH 4.0–6.0), 50 mM sodium phosphate (pH 6.5–7.5), 50 mM Tris-Cl (pH 8–9), and 50 mM glycine-NaOH (pH 10–11). In order to determine the optimal pH in the reductive direction, purified recombinant 20β-HSDH enzyme at a final concentration of 0.05 μM was added to 50 μM cortisol and 150 μM NADH in 500 μl buffer. The oxidation of NADH was measured continuously at 340 nM for 5 min by spectrophotometry and the initial activities were calculated. For the oxidative reaction, 0.05 μM of enzyme was added to 50 μM 20β-dihydrocortisol and 150 μM NAD^+^ in 500 μl buffer. The formation of NADH was measured continuously at 340 nm for 5 min and initial velocity was calculated.

### Phylogenetic analysis and bioinformatics

Candidate protein sequences for phylogeny reconstruction were retrieved from NCBI after BLAST using as query the short-chain dehydrogenase/reductase (SDR) family oxidoreductase (WP_051643274.1) from *B. desmolans* ATCC 43058. Seventy sequences with an identity cutoff of 30% with the 20β-HSDH were selected and aligned using MUSCLE v3.8.31 (13). The resulting alignment was manually examined and subsequent phylogeny reconstruction was performed with FastTree v2.1.7 (14) using the implemented Shimodaira-Hasegawa test to estimate the reliability of each split in the tree. iTOL (15) was used for visualization of the obtained phylogeny. Deduced protein molecular weight was determined using ExPASy toolkit (16).

## RESULTS

### *B. desmolans* expresses 20β-HSDH activity

**Fig. 1.**
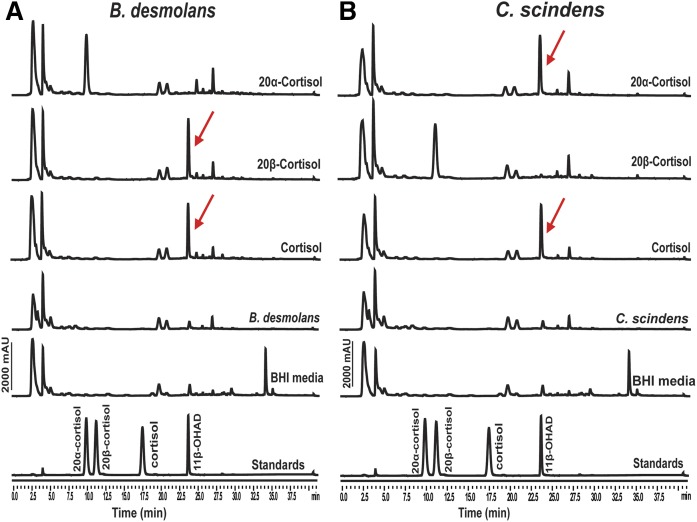
Confirmation of 20β- and 20α-HSDH activity from *B. desmolans* ATCC 43058 (A) and *C. scindens* ATCC 35704 (B). Each steroid substrate (50 μM) was incubated with both *B. desmolans* ATCC 43058 and *C. scindens* ATCC 35704 in BHI media for 48 h. The products were extracted and analyzed using HPLC equipped with a C-18 analytical column (Capcell Pak c18). Peak retention times and peak areas of samples were compared with standard steroid molecules. Red arrows denote the formation of 11β-OHAD from cortisol substrates.

Net conversion of cortisol to 11β-OHAD was observed in the presence of cortisol by both *C. scindens* ATCC 35704 and *B. desmolans* ATCC 43058 (**Fig. 1**). Net conversion of 20α-dihydrocortisol to 11β-OHAD was observed after 24 h growth by *C. scindens* ATCC 35704. By contrast, 20α-dihydrocortisol was not metabolized after 48 h by *B. desmolans* ATCC 43058. As expected, net conversion of 20β-dihydrocortisol to 11β-OHAD was observed in cultures of *B. desmolans* ATCC 43058. These results confirm that *B. desmolans* ATCC 43058 elaborates a 20β-HSDH, but lacks 20α-HSDH activity.

### Identification of the steroid-17,20-desmolase operon and prediction of a novel 20β-HSDH gene in *B. desmolans* ATCC 43058

The *desA*-deduced amino acid sequence (CLOSCI_00899) from *C. scindens* ATCC 35704 was used to query the nonredundant protein database (BLASTP). DesA is annotated in the thiamine-pyrophosphate-dependent enzymes superfamily domain, particularly N-terminal subunit transketolase (EC 2.2.1.1). The highest hits (E value 0.0, 2e^−178^) were against amino acid sequences encoded by *C. cadaveris* (WP_027640052) and *B. desmolans* (WP_031476417) with 82% identity/89% similarity and 81% identity/88% similarity, respectively (supplemental Fig. S1). A deduced amino acid sequence (WP_016455356.1) from the taxon, *P. lymphophilum* ACS-093-V-SCH5, shared 75% identity/86% similarity with DesA from *C. scindens* ATCC 35704.

**Fig. 2.**
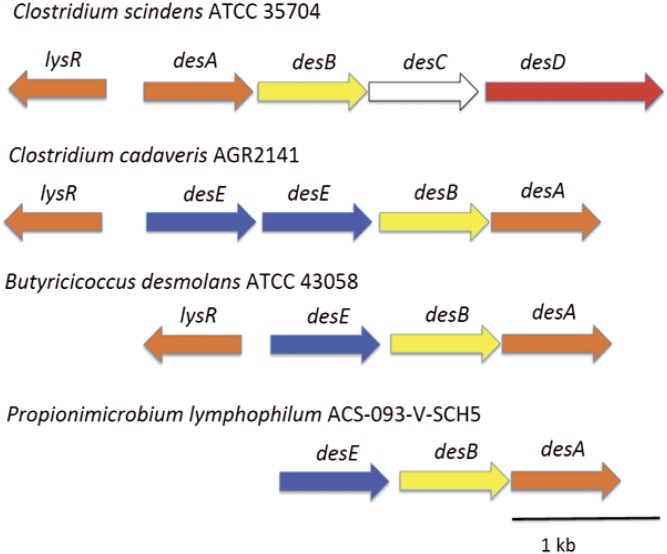
Identification and comparison of the desmolase operon of *B. desmolans* ATCC 43058 and *C. cadaveris* AGR2141 with *C. scindens* ATCC 35704. *lysR*, gene encoding putative LysR transcription factor; *desA* and *desB*, putative steroid-17,20-desmolase; *desC*, 20α-HSDH; *desE*, 20β-HSDH; *desD*, putative steroid transporter.

Further examination of the genes flanking *desA* revealed what appeared to be conserved *desB* gene in addition to an open reading frame named WP_051643274.1, whose gene product consisted of 282 amino acids containing the canonical Gly-X-X-X-Gly-X-Gly N-terminal pyridine nucleotide-binding motif typical of SDR family enzymes (supplemental Fig. S2) (17, 18). The deduced subunit molecular mass for the protein encoded by WP_051643274.1 is 30.29 kDa. Because *desC* from *C. scindens* ATCC 35704 encodes a 20α-HSDH with annotation in the L-threonine dehydrogenase family (EC 1.1.1.103) and *B. desmolans* and *C. cadaveris* are reported to express 20β-HSDH (10), we hypothesized that WP_051643274.1 encodes a 20β-HSDH. We are here referring to WP_051643274.1 as the *desE* gene. Comparison of the gene organization between *C. scindens*, *C. cadaveris*, *B. desmolans*, and *P. lymphophylum* is depicted in **Fig. 2**.

### Characterization of mRNA encoding *desEAB* operon in *B. desmolans*

To determine whether the *desEAB* genes are coexpressed as a polycistronic operon, we isolated total RNA from mid-log phase *B. desmolans* and designed oligonucleotides (supplemental Table S1) spanning the intergenic regions between *desE*-*desA* and *desA*-*desB*. Amplification from the cDNA demonstrates that the 20β-HSDH (*desE*) is coexpressed with the putative steroid-17,20-desmolase (*desAB*) (supplemental Fig. S3). The transcription initiation site was determined by SMARTER-RACE PCR to be an adenine residue at position −144. The putative ribosome-binding site (AGGAGGA) was identified starting at position −9. Conserved upstream elements (−149 to −182 and −183 to −196) were identified in alignments between the region between *lysR* and *desA* in *C. scindens* ATCC 35704 and *lysR* and *desE* from *C. cadavaris* AGR2141 and *B. desmolans* ATCC 43058 (supplemental Fig. S4).

### Cloning, expression, and purification of 20β-HSDH from *B. desmolans*

**Fig. 3.**
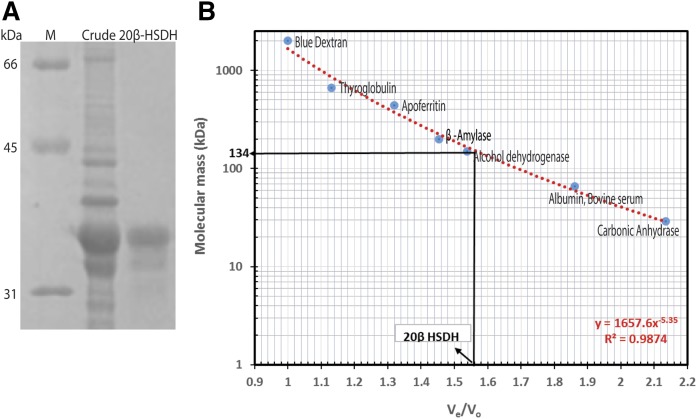
Purified 20β-HSDH from *B. desmolans* ATCC 43058. A: SDS-PAGE of crude and purified 20β-HSDH. Lane M, prestained molecular mass marker (Bio-Rad); lane 1, crude cell lysate; lane 2, 20β-HSDH purified by Strep-tag affinity chromatography. B: Native molecular size analysis of purified 20β-HSDH by size-exclusion chromatography.

To determine whether WP_051643274.1 encodes 20β-HSDH, the gene was cloned into pET51b(+) expression vector. The N-terminal streptavidin-tagged recombinant protein was purified by Strep-Tactin® affinity chromatography. The recombinant 20β-HSDH protein was resolved as a single band on SDS-PAGE with subunit molecular mass of 33.8 ± 3.7 kDa (**Fig. 3A**).

To determine the native molecular mass, the purified recombinant 20β-HSDH was separated by gel filtration chromatography and eluted at 1.56 V_e_/V_o_ corresponding to a relative molecular mass of 134 ± 4 kDa, eluting after yeast alcohol dehydrogenase (150 kDa) (Fig. 3B). The 20β-HSDH activity of protein fractions corresponding to eluted volume was confirmed with cortisol and NADH by standard spectrophotometry assay (data not shown). The gel filtration elution profile, coupled with the subunit molecular mass determined by SDS-PAGE, suggests a tetrameric structure (3.96) with subunits of identical molecular mass.

### Biochemical characterization of recombinant 20β-HSDH

**Fig. 4.**
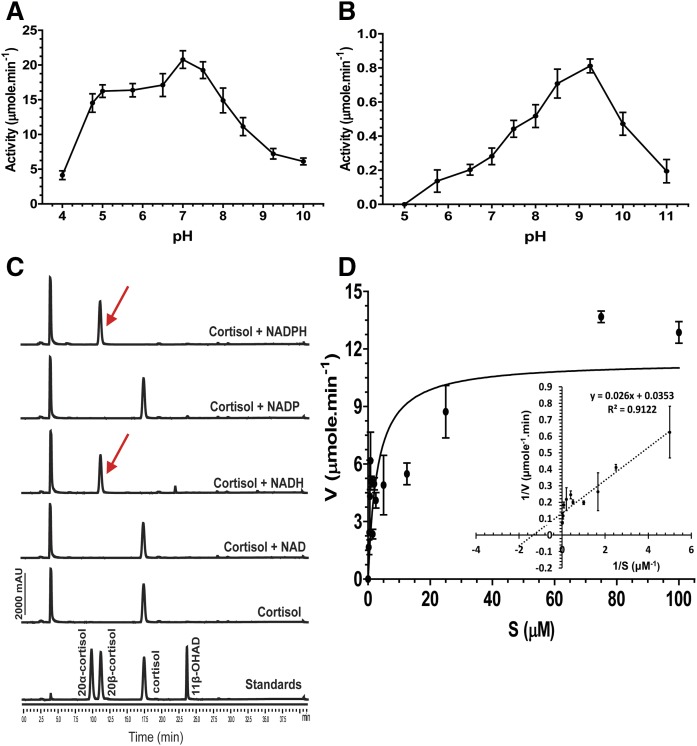
Biochemical characterization of recombinant 20β-HSDH from *B. desmolans* ATCC 43058. A: Effect of pH on reductive activity of 20β-HSDH. The 20β-HSDH (0.05 μM) was incubated with cortisol (50 μM) and NADH (150 μM) in sodium citrate buffer (pH 4–6), sodium phosphate buffer (pH 6.5–7.5), Tris-Cl buffer (pH 8–9), and glycine-NaOH buffer (pH 10) for 5 min. B: Effect of pH on oxidative activity of 20β-HSDH. The 20β-HSDH (0.05 μM) was incubated with cortisol (50 μM) and NAD^+^ (150 μM) in sodium citrate buffer (pH 5–6), sodium phosphate buffer (pH 6.5–7.5), Tris-Cl buffer (pH 8–9), and glycine-NaOH buffer (pH 10–11) for 5 min. One-way ANOVA followed by Tukey’s multiple comparison tests between the pH values using GraphPad Prism 5.04 (GraphPad Software Inc.). C: Cofactor requirement of 20β-HSDH activity from *B. desmolans*: 20β-HSDH (0.05 μM) was incubated with cortisol (50 μM) and respective pyridine nucleotides (150 μM) in sodium phosphate buffer (pH 7) overnight. D: Michaelis-Menten and Lineweaver-Burk plot of purified 20β-HSDH using cortisol as substrate.

The pH optima of the 20β-HSDH enzyme was determined in the oxidative and reductive directions by spectrophotometry. Steroid reduction was observed within the pH range 4.0–10, retaining 19.9 ± 3.1% of maximal activity at pH 4.0 and 29.5 ± 3.7% of maximal activity at pH 10.0 (**Fig. 4A**). Between pH 6.5 and pH 7.0 (82.6 ± 10.8% to 100%), we found a statistically significant difference and maximum activity was reached at around pH 7.0–7.5 (100% activity at pH 7.0 vs. 93.2 ± 7.6% at pH 7.5; *P* < 0.05).

Similarly steroid oxidation, as measured by the conversion of NAD^+^ to NADH in the presence of 20β-dihydrocortisol, was observed over a broad pH range, from pH 5.2 to 11.0 with maximal enzyme activity between pH 8.5 and 9.25 (Fig. 4B). Strikingly, even at maximal activity (0.81 ± 0.04 μmol·min^−1^), the oxidative direction represented only 3.9% of the maximal activity in the reductive direction (20.8 ± 1.2 μmol·min^−1^). These results indicate that under in vitro conditions, the enzyme catalyzes reductive reactions far more readily than oxidative reactions.

Next, we tested pyridine-nucleotide cofactor specificity in both the reductive and oxidative directions. When NADPH was used as a cosubstrate under optimum conditions for reduction of cortisol, the initial reaction rate observed was <5% of that with NADH, indicating that NAD(H) is the preferred pyridine nucleotide. Nevertheless, the purified enzyme (50 nM) catalyzed the net reduction of cortisol with either NADH or NADPH as the cosubstrate after 12 h incubation; however, formation of 20β-dihydrocortisol was not detected if NAD^+^ or NADP^+^ was added as cofactor, as expected (Fig. 4C). *K_m_* and *V_max_* values for cortisol in the presence of saturating NADH were measured from the linear portion of the Lineweaver-Burk plot (Fig. 4D). The calculated kinetic parameters are reported in **Table 1**.

**TABLE 1.** Kinetic parameters for cortisol and NADH with purified 20β-HSDH from *B. desmolans* ATCC 43058

Kinetic Parameters	Value
*K_m_* (μM)	0.80 ± 0.06
*V_max_* (μmol·min^−1^)	30.36 ± 1.97
*K_cat_* (μmol·μM^−1^·min^−1^)	607 ± 39
*K_cat_*/*K_m_*	760 ± 7.69

The substrate-specificity of recombinant 20β-HSDH is shown in **Table 2**. The equilibrium for recombinant 20β-HSDH clearly favors the reductive direction. Cortisol is the preferred substrate, as it was more rapidly converted to 20β-dihydrocortisol relative to other structurally similar steroids tested (Table 2). Substrates lacking 11β-hydroxy or 17α-hydroxy resulted, respectively, in 49.8% and 39.8% of the activity observed with cortisol. Interestingly, recombinant 20β-HSDH showed only 49.2% of the activity observed with cortisol when 5β-tetrahydrocortisol was substrate, suggesting recognition of the 3-hydroxy/keto and/or AB-ring structure. In the oxidative direction, 20β-hydroxy substrates ranged from no activity to 4.1% activity relative to cortisol, and recombinant 20β-HSDH was not active against 20α-hydroxy substrates.

**TABLE 2.** Substrate specificity of purified 20β-HSDH from *B. desmolans*

Steroids	Co-substrate	Activity (μmol/min)	Relative Activity (%)
4-pregnen-11β,17,21-triol-20,3-dione	NADH	21 ± 1.4	100
4-pregnen-17,21-diol-3,20-dione	NADH	10.5 ± 1.3	49.8
5β-pregnan-3α,11β,17,21-tetrol-20-one	NADH	10.4 ± 1.7	49.2
4-pregnen-11β,21-diol-3,20-dione	NADH	8.4 ± 1.3	39.8
4-pregnen-11β,17,20β,21-tetrol-3-one	NAD^+^	0.80 ± 0.02	4.1
4-pregnen-17,20β-diol-3-one	NAD^+^	0.43 ± 0.03	2.1
4-pregnen-11β,20β,21-triol-3-one	NAD^+^	NA	NA
4-pregnen-11β,17,20α,21-tetrol-3-one	NAD^+^	NA	NA
4-pregnen-17,20α-diol-3-one	NAD^+^	NA	NA

The reactions were initiated by addition of 50 nM purified enzyme to 0.5 ml of 0.05 M sodium phosphate buffer (pH 7) containing 0.15 M NaCl, 20% glycerol, 0.01 M 2-mercaptoethanol, 50 μM steroid, and 150 μM NAD(H) at 37°C. All values are the means of four replicates. NA, no activity.

### Phylogenetic analysis of 20β-HSDH

**Fig. 5.**
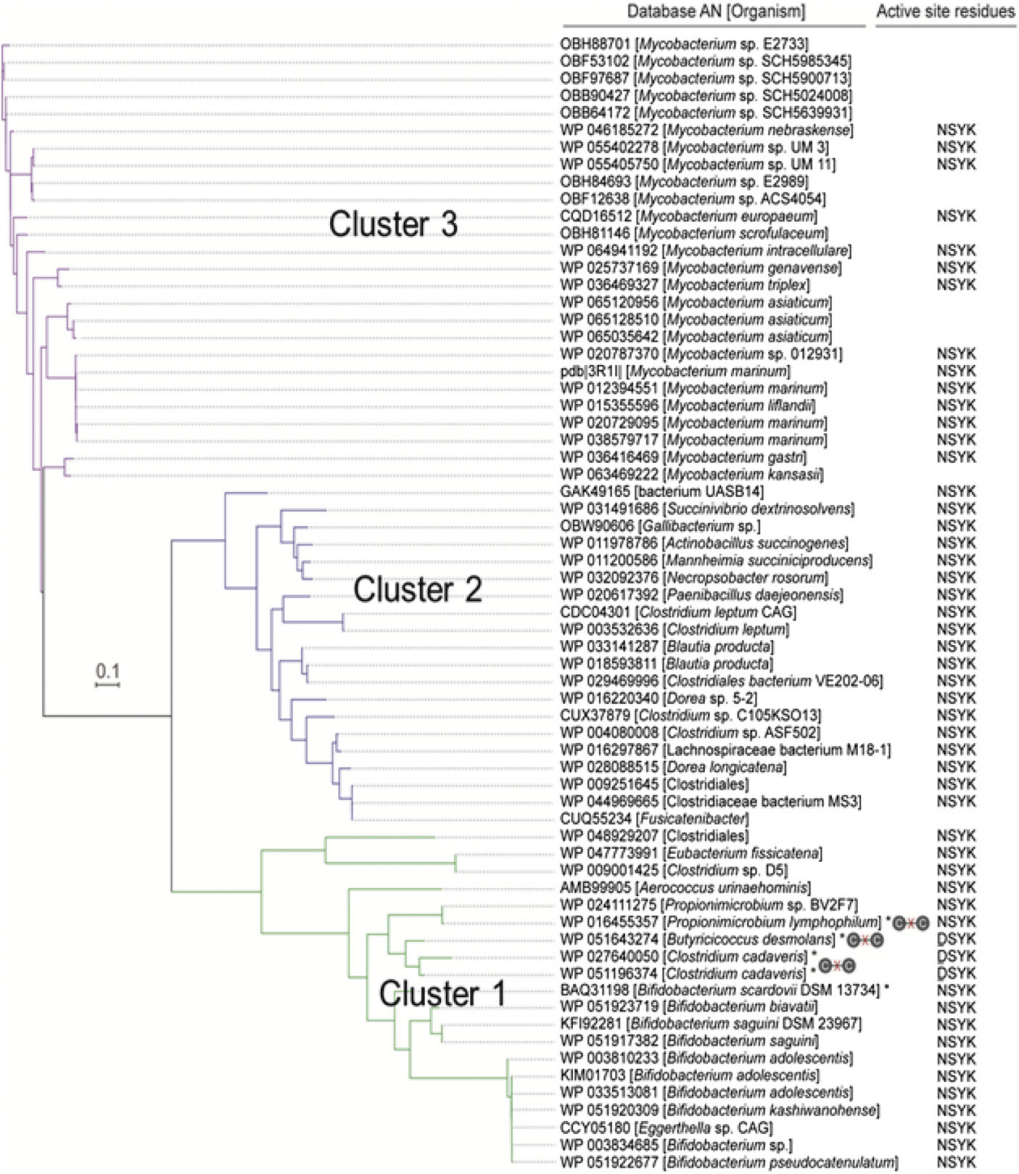
Phylogenetic analysis of identified 20β-HSDH. The amino acid sequences of candidate proteins with an identity cutoff of 30% retrieved from NCBI. The phylogenetic tree was reconstructed using FastTree v2.1.7. The active sites of 20β-HSDH of different organisms are mentioned on right side. Steroid-17,20-desmolase-expressing strains are denoted by an “x” through a C-C bond. Predicted active-site residues are depicted adjacent to strain designations.

Of the 70 sequences that made the 30% amino acid sequence identity cutoff with *desE*, the sequences formed three major clusters (**Fig. 5**), the most distantly related of which was composed exclusively of *Mycobacterium* *spp.* (cluster 3), followed by a cluster inhabited by anaerobic gut bacteria inhabiting the gastrointestinal tracts of ruminants and monogastric fermenters (cluster 2). Several of these genes are found in succinate-producing gram-negative ruminants (*Succinivibrio dextrinosolvens*, *Gallibacterium spp.*,* Actinobacillus succinogenes*, *Mannheimia succiniciproducens*). Several others belong to members of Clostridium cluster XVIa, including species of *Dorea* and *Blautia producta*, and Clostridium cluster IV, including *Clostridium leptum*. Notably, a conserved active-site tetrad, NSYK, was identified in the majority of sequences within each cluster, with the exception of *B. desmolans* and *C. cadaveris*, whose active-site residues, according to the multiple-sequence alignment, are DSYK (Fig. 5; supplemental Fig. S2).

Proteins from *C. cadaveris* are represented twice in cluster 1 (encoded by WP_027640050, WP_051196374), and most closely related to the 20β-HSDH from *B. desmolans*. Examination of their genomic location revealed that the two putative 20β-HSDH encoding genes share 91% amino acid sequence similarity, suggesting the likelihood of gene duplication (Fig. 2). The two *desE* genes in *C. cadaveris* are located adjacent to *desAB* genes. Closely related to the *desE* amino acid sequence from *B. desmolans* and *C. cadaveris* are SDR-family proteins from two strains of *P. lymphophilum*, which are detected in urine and have been isolated (Fig. 5). Like *B. desmolans* and *C. cadaveris*, the genes encoding putative 20β-HSDH from both strains of *P. lymphophilum* are adjacent to *desAB* genes, suggesting that these bacterial strains are capable of converting cortisol to 11β-OHAD.

Cluster 1 also contains several species of bifidobacteria. The *desAB* genes were not found in the available genome sequences of the bifidobacteria species represented in the phylogeny, suggesting that these strains may harbor 20β-HSDH, but not steroid-17,20-desmolase activity. Indeed, *desAB* genes were not found in any other members of clusters 1, 2, and 3, except for *B. desmolans*, *C. cadaveris*, and *P. lymphophilum*.

### Verification of functional steroid metabolic activity by selected members of cluster III of the 20β-HSDH phylogeny

**Fig. 6.**
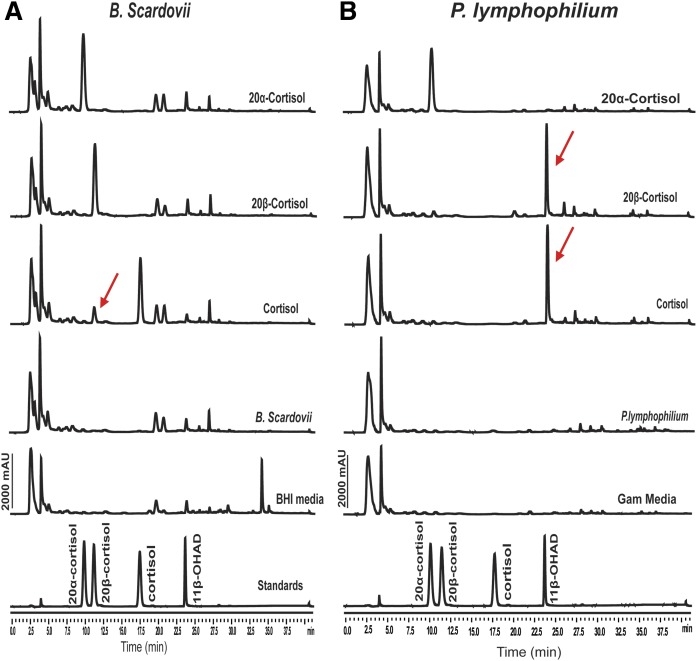
Confirmation of 20β-HSDH activity from *B. scardovii* ATCC BAA-773 (A) and steroid-17,20-desmolase activity in *P. lymphophilum* ACS-093-V-SCH5 (B). Each steroid substrate (50 μM) was incubated with both *B. scardovii* in BHI medium and *P. lymphophilum* in Gifu anaerobic medium for 48 h. The products were extracted and analyzed using HPLC equipped with a C-18 analytical column (Capcell Pak c18). Peak retention times and peak areas of samples were compared with retention times of standards.

We next sought to determine whether two organisms, whose gene products cluster closely with the 20β-HSDH from *B. desmolans* ATCC 43058, exhibited 20β-HSDH activity. We tested *B. scardovii* ATCC BAA-773 and *P. lymphophilum* ACS-093-V-SCH5 for 20β-HSDH and steroid-17,20-desmolase activity, the latter strain hypothesized to express both activities because it encodes the *desABE* operon (Fig. 2). Neither organism was capable of metabolizing 20α-dihydrocortisol. We detected the formation of 20β-dihydrocortisol when cortisol was the substrate; however, we did not detect the oxidation of 20β-dihydrocortisol by whole-cells of *B. scardovii*, which is consistent with the observation that, at equilibrium, the recombinant 20β-HSDH from *B. desmolans* converted ∼98% of cortisol to 20β-dihydrocortisol in vitro over a 24 h period. We did not detect the formation of 11β-OHAD by cultures of *B. scardovii* ATCC BAA-773. The 20β-HSDH and steroid-17,20-desmolase activity were confirmed in *P. lymphophilum* ACS-093-V-SCH5 by the fact that only 20β-hydroxy substrate was side-chain cleaved, forming 11β-OHAD (**Fig. 6**).

## DISCUSSION

HSDH enzymes play an important role in steroid and bile acid metabolism for gut bacteria. *C. scindens* ATCC 35704 expresses 20α-HSDH and steroid-17,20-desmolase, but also 3α-HSDH and 7α-HSDH as part of the complex bile acid 7α-dehydroxylation pathway (19). The 7α-HSDH may serve two functions: *1*) convert 7-oxo-bile acids generated by other gut bacteria (20) to 7α-hydroxy bile acids, which can enter the bile acid 7α-dehydroxylation pathway; and *2*) regulate the flux of bile acids into the 7α-dehydroxylation pathway by reversible oxidation-reduction of the 7α-hydroxy group (19). Gut bacteria, in human and other mammalian hosts, are also capable of forming 7β-hydroxy bile acids, including, but not limited to, *Clostridium baratii* (21), *B. producta* (22), and *Ruminococcus gnavus* (23). While lacking a 7β-HSDH, necessary to epimerize the 7-hydroxy, *C. scindens* and other bile acid 7-dehydroxylating bacteria circumvent this need by expressing a 7β-dehydratase (20). Interestingly, epimerization of the 3α-hydroxy group to 3β-hydroxy “iso-bile acids” (24) prevents 7α-dehydroxylation (J. M. Ridlon and P. B. Hylemon, unpublished observations). *C. scindens* lacks 3β-HSDH, and the first enzymatic step following bile acid CoA-ligation is oxidation of the 3α-hydroxy group (20).

Steroid-17,20-desmolase and 21-dehydroxylation of glucocorticoids are similar to 7α-dehydroxylation of bile acids in that certain bacterial modifications of steroid substrates preclude these pathways. Namely, steroid-17,20-desmolase requires a 20-keto group on 17α,21-dihydroxy corticosteroids (6). *C. scindens* ATCC 35704 is capable of converting 20α-dihydrocortisol, but not 20β-dihydrocortisol, to 11β-OHAD due to expression of 20α-HSDH, but lacking a gene encoding 20β-HSDH, as we and others (6) have demonstrated. The reverse is true of *B. desmolans*, *C. cadavaris* (10), and *P. lymphophilum*. The rate of 21-dehydroxylation of cortisol, corticosterone, or deoxycorticosterone to progestin-derivatives by *Eggerthella lenta* may be altered by gut bacterial conversion of 20-keto to 20-hydroxy because *E. lenta* lacks 20α-HSDH and 20β-HSDH activity, and 21-dehydroxylase requires 20-keto substrates (25, 26). By working out the biochemical pathways in steroid and other microbial metabolic pathways, it may be possible to alter the composition of steroids and bile acids through probiotics and/or develop specific inhibitors of particular bacterial steroid modifying enzymes, resulting in a measurable alteration in the metabolomic profile and phenotypic effects on the host.

Winter et al. (27) isolated a strain of *Bifidobacterium adolescentis* from human feces that displayed 20β-HSDH activity. Phylogenetic analysis of the *desE* gene in *B. desmolans* resulted in identification of several *Bifidobacterium spp.* strains, including *B. adolescentis*, encoding *desE* but lacking *desAB*. This is consistent with the observation that *B. scardovii* ATCC BAA-773 produces 20β-dihydrocortisol from cortisol (Fig. 6). The observation that some bifidobacteria strains encode and express 20β-HSDH, but not steroid-17,20-desmolase, is interesting in this regard. It is possible that 20β-HSDH activity in bifidobacteria may reduce the conversion of cortisol to 11β-OHAD in GI tracts dominated by 20α-HSDH-elaborating bacteria, such as *C. scindens* ATCC 35704.

Metabolites of gut bacterial steroid-17,20-desmolase and steroid 21-dehydroxylase are potent inhibitors of host 11β-HSDH isoform 2 (11β-HSD2) (28–31). The absorption of 11β-hydroxyandrogens and 11β-hydroxyprogestins from the GI tract into the host may have varied effects depending on the target tissue. Inhibition of 11β-HSD2 in colonic epithelium may be protective against colorectal cancer (32). However, inhibition of 11β-HSD2 in vascular tissue (also by inhibition of 11β-HSD1) and kidney is a possible contributing factor in essential hypertension (33). A current method of determining the functionality of 11β-HSDH isoforms in patients is urinary measurement of the cortisol:cortisone ratio and potassium levels (33).

Of particular note is the observation that some strains of *P. lymphophilum*, which may be members of the normal urinary microbiota (34, 35), previously unrecognized to encode steroid-17,20-desmolase and 20β-HSDH, have been shown in the current study to convert cortisol to 11β-OHAD. This could potentially be of significance given that renal activity of 11β-HSDH-2 is currently measured by the ratio of cortisol:cortisone. However, if significant metabolism of either metabolite or both in the bladder occurs in some individuals, precise measurement of this ratio may not reflect accurate renal activity of this enzyme, and consideration of the urinary microbiome may be necessary.

**Fig. 7.**
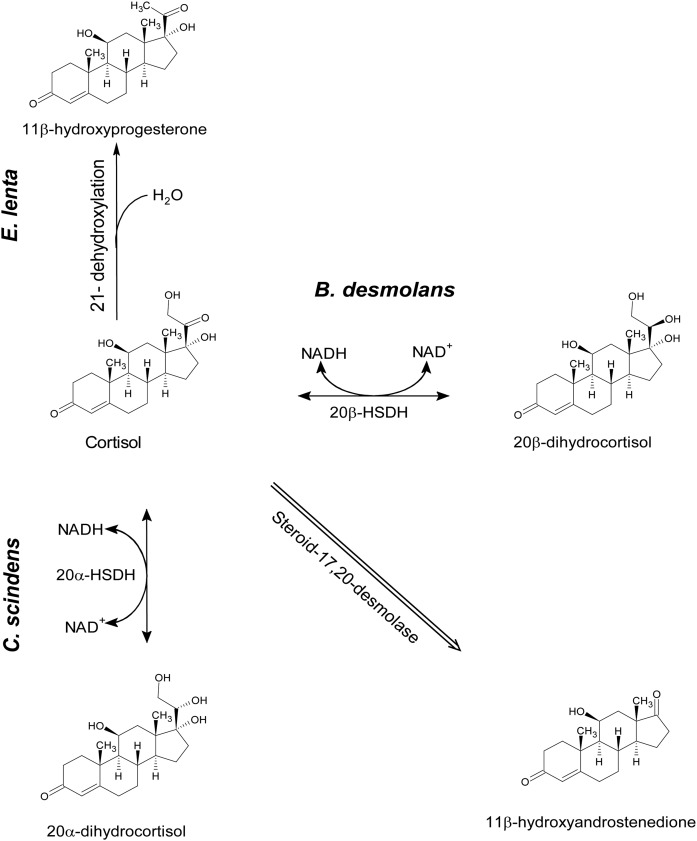
Reactions in the steroid-17,20-desmolase and 21-dehydroxylase pathways by gut bacteria.

Finally, our phylogenic analysis of *desE* also included numerous genes from *Mycobacterium spp.* (**Fig. 7**), including *M. tuberculosis* (supplemental Fig. S5). Mycobacteria encode numerous genes involved in the catabolism of host fatty acids, cholesterol, and oxysterols for the generation of energy and mycolic acid (36, 37), but also endocrine disruption by altering the structure of host immune signaling steroids (38). A 3α,20β-HSDH (Rv2002) has been reported in *M. tuberculosis*, which is suggested to be involved in steroid metabolism (39). Rv2002 did not show up in our phylogeny (Fig. 5) or in our extended phylogeny (supplemental Fig. S5), which included several *M. tuberculosis* SDR-family proteins. It is possible that the proteins identified in our phylogeny are utilized by mycobacteria to metabolize host sterols or perhaps environmental sterols from plants and animal material; however, this remains the subject of future investigation.

## Supplementary Material

Supplemental Data
