# Supplementary material for: Identification and characterization of a 20β-HSDH from the anaerobic gut bacterium Butyricicoccus desmolans ATCC 43058
Source: J Lipid Res. 2017 Apr 28;58(5):916–25. doi: 10.1194/jlr.M074914 (PMC5408610; doi:10.1194/jlr.M074914)
Supplement: Supplemental Data [file supp_58_5_916__index.html]

Identification and characterization of a 20β-hydroxysteroid dehydrogenase from the anaerobic gut bacterium Butyricicoccus desmolans ATCC 43058 — Identification and characterization of a 20β-HSDH from the anaerobic gut bacterium Butyricicoccus desmolans ATCC 43058 — Supplemental Data 

# Identification and characterization of a 20β-HSDH from the anaerobic gut bacterium *Butyricicoccus desmolans* ATCC 43058

## Supplemental Data

- Supplemental Material (.docx, 1.4 MB) - Supplemental Material
