# Supplementary material for: Identification and characterization of a 20β-HSDH from the anaerobic gut bacterium Butyricicoccus desmolans ATCC 43058
Source: J Lipid Res. 2017 Apr 28;58(5):916–25. doi: 10.1194/jlr.M074914 (PMC5408610; doi:10.1194/jlr.M074914)
Supplement: Supplemental Data [file 10.1194_M074914_jlr.M074914-1.docx]

**Supplementary Information**

**Identification and Characterization of a 20β-hydroxysteroid dehydrogenase from the anaerobic gut bacterium *Butyricicoccus desmolans* ATCC 43058**

Saravanan Devendran^a,b^, Celia Méndez-García^b^ and Jason M. Ridlon^a,b,c^

Department of Animal Sciences^a^, Carl R. Woese Institute for Genomic Biology^b^, Division of Nutritional Sciences^c^, University of Illinois at Urbana-Champaign, Urbana, Illinois 61801, USA

Supplementary Table S1: **Primers used in cloning of *B. desmolans* 20β-hydroxysteroid dehydrogenase, intergenic PCR and 5’-RACE PCR.**

| Gene | Forward primer |
| --- | --- |
|  | Reverse primer |
| 20β-HSDH | 5'-ATATATGGATCCATGGCAGAAGAATTCTACGCAGTATATCC-3' |
|  | 5'-ATATATAAGCTTTTAGAAGATCGAATAGCCGCCGTC-3' |
| DesEA | 5’-ATTTCCCCGGGCTATATCTG-3’ |
|  | 5’-ACCAAATCGCGTACGTTTTC-3’ |
| DesAB | 5’-AGACACTGGCCAATTTACCG-3’ |
|  | 5’-CCGCAGCAAAAAGTAAATCC-3’ |
| RACE PCR (longer primer) | 5’-GATTACGCCAAGCTTCCATTACGCCTTCGTGGATGCCCGACC-3’ |
| RACE PCR(short primer) | 5’-GATTACGCCAAGCTTCCGTGCCCAGTTCGCGGATCAAATCGTC-3’ |

**Supplemental Figures**

Supplemental Figure S1.
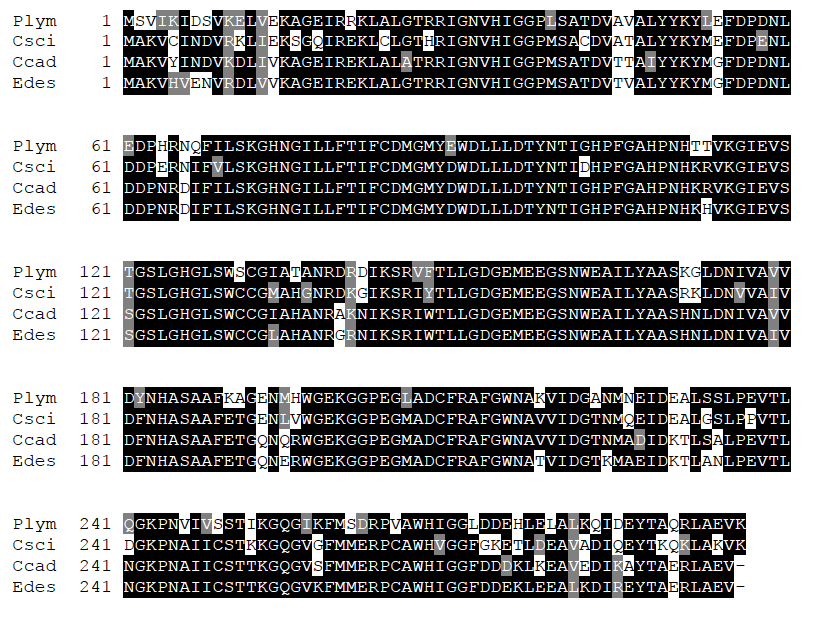


Supplemental Figure S1: **Multiple sequence alignment of the amino acid sequences of DesA from various bacteria**. The sequences were aligned with ClustalW (http://www.ebi.ac.uk/Tools /Clustalw). The shaded residues are conserved amino acids among different bacterial HSDHs. Plym, *Propionimicrobium lymphophilium*, Csci, *Clostridium scindens* ATCC 35704, Ccad, *Clostridium cadaveris* and Edes, *Butyricicoccus desmolans*.

Supplemental Figure S2.

Supplemental Figure S2 cont.

Supplemental Figure S2: **Multiple sequence alignment of amino acid sequences of 20β- hydroxysteriod dehydrogenases from various bacteria.** The sequences were aligned with ClustalW (http://www.ebi.ac.uk/Tools /Clustalw). The shaded residues are conserved amino acids among different bacterial HSDHs. 3,20strepexfol, 3α, 20β-HSDH from *Streptomyces hydrogenans.* BifidopseudoSDR, short chain dehydrogenase/reductase (SDR) from *Bifidobacterium pseudocatenulatum*. BifidokashiSDR, SDR from *Bifidobacterium kashiwanohense*, BifidoadolSDR, SDR from *Bifidobacterium adolescentis*. BifidosaguiSDR, SDR from *Bifidobacterium saguini*. Bifidoscardo98 and Bifidoscardo36 and BifidoscarSDR, SDRs from *Bifidobacterium scardovii*. PropionBV2f7SDR, SDR from *Propionimicrobium sp*. PropionlymphoSD, SDr from *Propionimicrobium lymphophilum*. Closeadav50, and Closeadav71, SDR from *Clostridium sp*. Butyrdesmo, SDR from *Butyricicoccus desmolans*. MycomariSDR95 and MycomariSDR51, SDR from *Mycobacterium marinum*. MyconebraskSDR, SDR from *Mycobacterium nebraskense*. MycogastriSDR, SDR from *Mycobacterium gastri*, SuccindextriSDR, SDR from *Succinivibrio dextrinosolvens*. BlautprodSDR, SDR from *Blautia producta*.

Supplemental Figure S3.


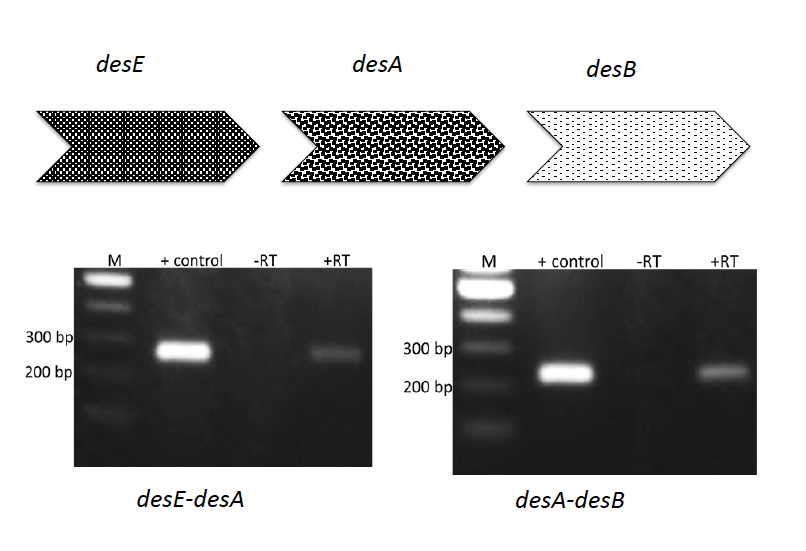


Supplemental Figure S3: **RT-PCR of intergenic regions of the desEAB operon from *B. desmolans* and RACE-PCR Gel**. RNA was isolated from CA induced cells. “+ “represents positive control which is PCR of *B. desmolans* genomic DNA. -RT is the negative control reaction in which cDNA synthesis lacks reverse transcriptase. Organization of genes is shown above for reference, blue arrows indicate positions of intergenic oligo binding.

Supplemental Figure S4.

Supplemental Figure S4: **Identification of the transcription initation site (TIS) upstream of the *desEAB* operon from *B. desmolans* 43058 and conserved UP elements conserved among steroid-17,20-desmolase expressing gut bacteria.** A. Agarose gel of RACE PCR product. TIS was located at reside -144 from the start codon of *desE* in *B. desmolans* ATCC 43058. A putative ribosome binding site (AGGAGGA) was located upstream of the start codon. B. Boxshade representation of CLUSTAL Omega alignment of the intergenic region between the *lysR* and the *desE/desA* genes. Represented in the alignment are *B. desmolans* (Bdes), *Clostridium scindens* (Csci), and *Clostridium cadaveris* (Ccad). Conserved regions corresponding to *B. desmolans* -149 to -182 and -183 to -196 were identified.

Supplemental Figure S5.


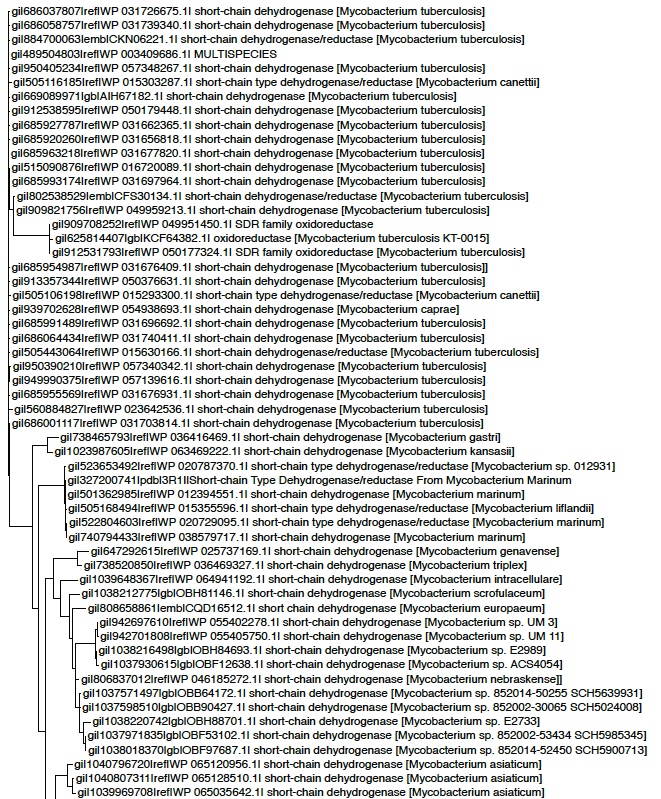


Supplemental Figure S5: **Extended Cluster III phylogeny including *Mycobacterium tuberculosis* proteins**.
